# Smoke pollution must be part of the savanna fire management equation: A case study from Darwin, Australia

**DOI:** 10.1007/s13280-022-01745-9

**Published:** 2022-05-24

**Authors:** Penelope J. Jones, James M. Furlaud, Grant J. Williamson, Fay H. Johnston, David M. J. S. Bowman

**Affiliations:** 1 Menzies Institute for Medical Research, University of Tasmania, 17 Liverpool St, Hobart, TAS 7000 Australia; 2 School of Natural Sciences, University of Tasmania, Private Bag 55, Hobart, TAS 7001 Australia; 3 Public Health Services, Department of Health, 17 Liverpool St, Hobart, TAS 7000 Australia

**Keywords:** Carbon abatement, Fire, Northern Australia, Particulate pollution, Smoke pollution, Tropical savanna

## Abstract

Savanna fire management is a topic of global debate, with early dry season burning promoted as a large-scale emissions reduction opportunity. To date, discussions have centred on carbon abatement efficacy, biodiversity and cultural benefits and/or risks. Here we use a case study of Darwin, Australia to highlight smoke pollution as another critical consideration. Smoke pollution from savanna fires is a major public health issue, yet absent so far from discussions of program design. Here, we assess the likely impacts of increased early dry season burning on smoke pollution in Darwin between 2004 and 2019, spanning the introduction and expansion of carbon abatement programs. We found increased smoke pollution in the early dry season but little change in the late dry season, contributing to a net annual increase in air quality standard exceedances. Geospatial analysis suggests this relates to increased burning in the path of early dry season trade winds. This study highlights the complex health trade-offs involved with any large-scale prescribed burning, including for carbon abatement.

**Supplementary Information:**

The online version contains supplementary material available at 10.1007/s13280-022-01745-9.

## Introduction

Savanna fire management is currently a topic of substantial global interest and debate. Much of this interest stems from the carbon abatement potential of prescribed burning programs that shift savanna burning from the late to the early dry season (Lipsett-Moore et al. 2018; Edwards et al. 2021; Laris 2021; Russell-Smith et al. 2021). Given that savanna fires contribute some 62% of global fire emissions annually (van der Werf et al. 2017), if effective, the emissions reductions could be substantial. In Australia, savanna burning programs for carbon abatement were developed in the mid-2000s and integrated into the carbon market with apparent success (Russell-Smith et al. 2015; Edwards et al. 2021). On this basis, it has been proposed that a substantial opportunity exists to implement carbon-marked-based savanna burning programs across other regions of the globe (Lipsett-Moore et al. 2018; Russell-Smith et al. 2021).

Without discounting the potential value of emissions reductions on a substantial scale, as with any landscape-scale intervention, the collateral benefits and/or risks of savanna burning for carbon abatement must be carefully factored into program implementation and design. To date, discussions of the impact, appropriateness and design of savanna burning programs have focused on carbon abatement efficacy (Laris 2021), emission measurement methodologies (Perry et al. 2020; Laris 2021), biodiversity (Perry et al. 2016; Corey et al. 2019), and economic and cultural benefits and/or risks (Russell-Smith et al. 2013, 2015, 2017; Ansell et al. 2020).

One critical factor—smoke pollution—has been notably absent. Smoke pollution from landscape fires is a globally significant public health problem (Johnston et al. 2012) and this is particularly true for the highly fire-prone savanna biome. Indeed, global analysis found that the greatest burden of disease from landscape fire smoke is associated with fires in tropical rainforests and savannas throughout the world, particularly in developing nations where savannas support substantial human populations (Johnston et al. 2012).

In this context, there is a clear need to consider the smoke pollution consequences of savanna burning programs, yet no studies have yet considered this relationship. One outcome of increased savanna burning for carbon abatement may be reduced smoke pollution, given the expected correlation between greenhouse gas and particulate emissions (Andreae 2019). This could be a valuable co-benefit. However, we argue that reduced smoke pollution exposure (and hence health costs) should not be assumed: the trade-offs between smoke exposure from prescribed and wildfire can be complex (Williamson et al. 2016a) and factors such as weather patterns and fuel conditions may impact smoke exposure patterns in unexpected ways.

In this paper we use Darwin, Australia as a case study with which to investigate the relationship between savanna fire regime change and smoke pollution, over a period spanning the introduction and expansion of early dry season burning programs for carbon abatement. Darwin offers an ideal natural experiment with which to consider this problem for several reasons. First, its smoke pollution problem is substantial. Surrounded by vast and largely uncleared *Eucalyptus* savannas, the city suffers severe smoke pollution every dry season, recording regular exceedances of the Australian air quality standard for 24-h average concentrations of particulate matter less than 25 μm in diameter (PM_2.5_) (25 µg m^3^) (Lorelei et al. 2020). The health impacts of this pollution are known to be substantial, including increased risks of asthma emergency department presentations and hospital admissions for respiratory and cardiovascular diseases (Johnston et al. 2002; Hanigan et al. 2008; Crabbe 2012), and disproportionately impact Indigenous peoples (Hanigan et al. 2008). Second, Darwin’s savanna fire pollution, including that derived from prescribed burning, can be traced with very little confounding: with little traffic or industrial pollution, almost all (95%) of Darwin’s particulate pollution is attributable to landscape fires (Denlay et al. 2001), and almost all early dry season fire is attributable to prescribed burning practices (Russell-Smith et al. 2020). Finally, Darwin offers a robust historical baseline in which to contextualise the nexus between fire management, fire activity and smoke pollution (Bowman et al. 2007b).

We exploit these characteristics to consider the relationship between prescribed burning and smoke pollution in Darwin over the period 2004–2019. Over this period there has been a substantial increase in early dry season burning linked to financial incentives for greenhouse gas abatement. In 2006, the Western Arnhem Land Fire Abatement Program began as a carbon abatement enterprise based on early dry season prescribed burning (Russell-Smith et al. 2013); in 2012, the Australian Government introduced early dry season savanna burning as a certified emissions reduction mechanism under its Carbon Farming Initiative (currently known as the Emissions Reduction Fund, ERF). The underlying premise is that early dry season burning releases fewer emissions than late dry season burning, because the fuel is moister and weather conditions milder—hence fires will be less extensive, combustion will less complete, and a smaller amount of fuel will be pyrolised (Russell-Smith et al. 2013; Edwards et al. 2021). By shifting burning from the late to the early dry season, emissions can thus be reduced on a net annual basis. Since its introduction, there has been a steady increase in the number of carbon abatement projects registered under the ERF, and these programs now cover approximately 25% of Australia's 1.2 million km^2^ tropical savanna biome (Edwards et al. 2021). This includes 55.5% of the land within a 500 km radius of Darwin (Australian Government Clean Energy Regulator 2021). Within this 500 km radius, 48% of land area under carbon abatement projects was indigenous land, 31% was pastoral land and 21% was conservation land (Australian Government Clean Energy Regulator 2021).

In this study we seek to establish whether the marked increase in early dry season prescribed burning between 2004 and 2019, increasingly motivated by carbon abatement schemes, has influenced smoke pollution over Darwin. Although it is a localised study, we seek to (i) raise the profile of this issue and (ii) demonstrate an approach to assessing smoke pollution impacts with applicability to other contexts in which prescribed burning, particularly burning for carbon abatement, is driving savanna fire regime change. Using statistical modelling that considers interannual, seasonal and daily meteorological variability and fire activity we test the following expectations:

1. Net annual smoke pollution in Darwin has declined in line with lower greenhouse gas emissions from regional savanna fires.
2. On a seasonal basis, smoke pollution in Darwin has shifted from the late to early dry season in response increased prescribed burning across a range of land tenures.

## Materials and methods

In order to address the research questions articulated above, we first assessed PM_2.5_ concentrations in Darwin over the period 2004–2019. We then conducted a parallel analysis of fire activity over all land within a 500 km radius. Finally, we used a novel combination of geospatial and statistical modelling techniques to assess the linkages between pollution, meteorology and fire activity trends.

### Study area

**Fig. 1:**
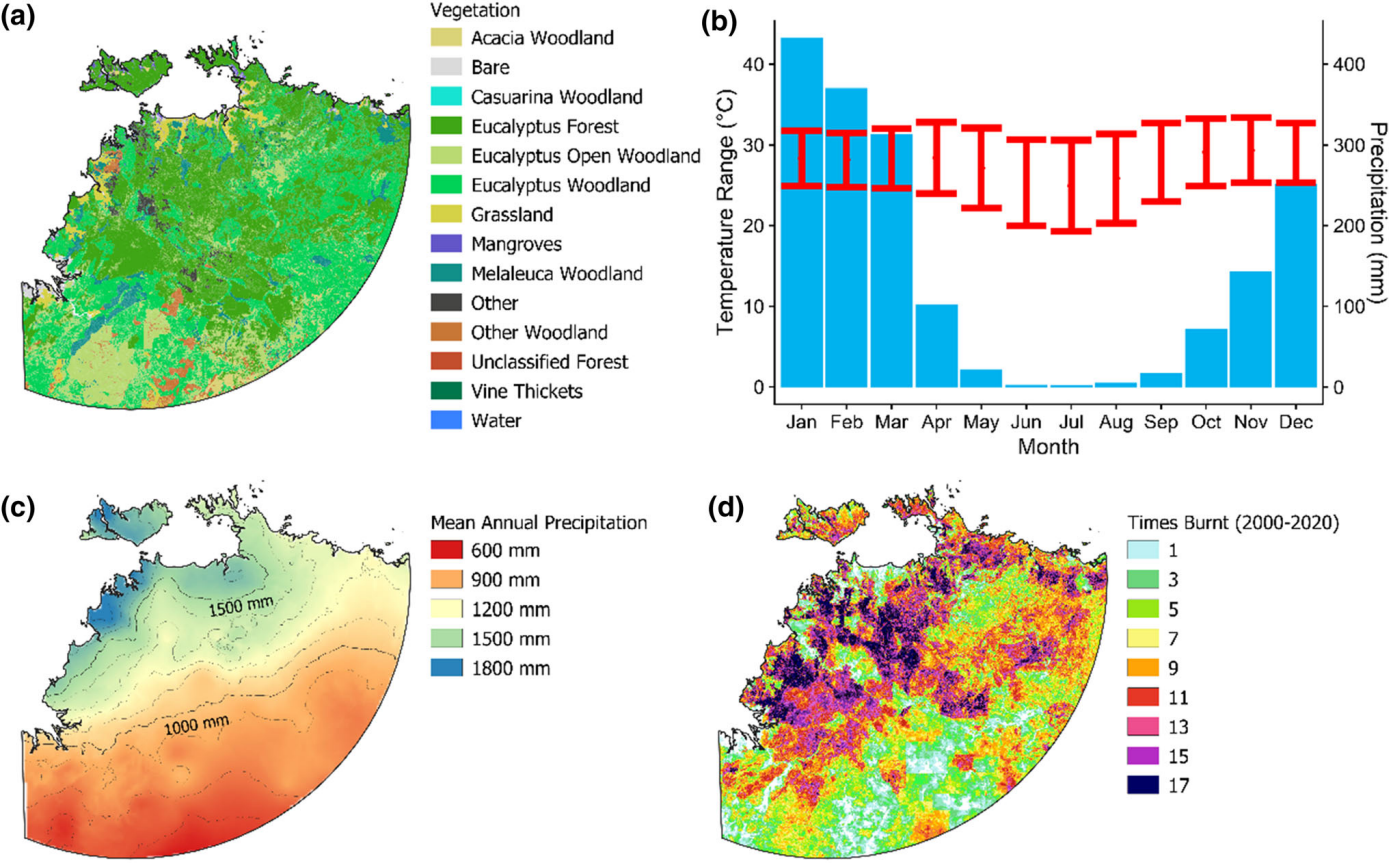
Vegetation, climate and fire characteristics of the study region, defined as all land within a 500 km radius of Darwin, Australia. Panel **a** Distribution of major vegetation types; the vegetation types that collectively comprise the savanna (eucalyptus forest, open woodland and woodland) cover 83% of the study region. Panel **b** Monthly temperature (red bars) and rainfall (blue columns) for Darwin. Panel **c** Mean annual rainfall, which decreases from north to south. Panel **d** Number of times burnt 2000–2020. Vegetation data were obtained from National Vegetation Inventory System 5.1 (Australian Government 2020). Precipitation data were sourced from WorldClim (Fick and Hijmans 2017). Fire history data were obtained from the North Australia and Rangelands Fire Information fire scar dataset (Jacklyn 2018)

Darwin (pop. 158 000) is the capital of Australia’s Northern Territory. As shown on Fig. 1a, the region is dominated by tropical open *Eucalyptus* forests and woodlands, collectively known as savanna. The climate is monsoonal: conditions are hot, wet and humid in the summer (November/December to March/April), and hot and dry for the rest of the year (Fig. 1b). Mean annual rainfall declines from approximately 1700 mm on the coast to 500 mm inland (Fig. 1c).

These climatic conditions promote high fire frequency: grasses and shrubs grow quickly in the wet season and cure during the dry season to form a continuous layer of fine surface fuel (Pausas and Ribeiro 2013). This pattern of intermediate and strongly seasonal productivity is the core factor that renders savanna systems the most flammable of any global biome (Pausas and Ribeiro 2013). Grass fuels are abundant, comprising nearly 90% of fine fuel biomass (Bowman et al. 2007a). As shown in Fig. 1d, many parts of the region burn with a return interval of 1–2 years; almost all fires occur in the dry season, although the proportion of fires that occur in the early vs late dry season varies regionally due to both climatic and land management factors (Russell-Smith et al. 2020). A significant portion of the study area is Aboriginal freehold land (see Fig. S1); there are also large areas protected for conservation, most notably Kakadu National Park. Pastoral land covers approximately 35% of the study area, mostly to Darwin’s south and west (Fig. S1).

### Data sources

We sourced daily average PM_2.5_ data from four monitoring stations across Darwin (Fig. S2). Casuarina operated from April 2004 to December 2011, Palmerston from January 2011 to December 2019, Stokes Hill from July 2017 to December 2019, and Winnellie from January 2013 to December 2019. Data were sourced directly from the Northern Territory Environment Protection Agency. No data were available for the period Oct–Dec 2012.

Weather data were derived from modelled 12 km historical weather grids (Su et al. 2019).

We focused on wind speed, wind direction, a continuous Haines Index (a measure of atmospheric instability), and the previous year’s precipitation, as these variables have been shown to strongly influence the effect of landscape fire on smoke pollution (Price et al. 2012). We extracted wind speed, wind direction, temperature and relative humidity for central Darwin and used these to calculate the Haines Index (Mills and McCaw 2010); total precipitation (mm) was derived across the study region for each antecedent wet season (defined as Nov–April).

Fire activity data were sourced from two products derived from the Moderate Resolution Imaging Spectroradiometer (MODIS) in order to combine the strengths of the two datasets. First, we extracted monthly fire extent data from the North Australia and Rangelands Fire Information fire scar dataset (Jacklyn 2018) and used these to calculate monthly, seasonal and annual area burnt. Second, we extracted thermal anomaly data collected by MODIS twice daily at 1 km resolution (NASA FIRMS 2020). This method registers map pixels with a substantially higher temperature than their surrounding environment as having an active fire, referred to as a ‘hotspot’ (Justice et al. 2002). While hotspot data provide a less accurate metric than area burnt, data are available to support daily analysis and each hotspot is ascribed a Fire Radiative Power (FRP) metric. We used FRP as our measure of fire intensity (Wooster et al. 2005), which has been demonstrated to be a good predictor of smoke pollution (Price et al. 2012).

Finally, we obtained land tenure and land use data for land within 500 km of Darwin from the Northern Territory Department of Infrastructure, Planning, and Logistics (Staben and Edmeades 2017).

### Data analysis

All data analysis was performed in R version 3.5.5 (R Core Team 2020) using the packages sf, geosphere, and openair (Carslaw and Ropkins 2012; Karney 2013; Pebesma 2018).

First, we generated a daily time series of PM_2.5_ for Darwin by taking the maximum daily (24-h) average PM_2.5_ reading recorded at any of the stations that were operational on a given day. Daily average PM_2.5_ was closely correlated across the stations (Fig. S2).

We then used this dataset to assess temporal trends in smoke pollution over the 2004–2019 study period. Trends were assessed in two separate smoke pollution metrics: (i) daily average PM_2.5_; and (ii) the number of exceedances of the Australian 24-h air quality standard (25 µg/m^3^). Similar to Williamson et al. (2012), we used generalised linear models (GLMs) to assess pollution trends, specifically gamma GLMs for PM_2.5_ and Poisson GLMs for exceedances. We tested trends on an annual, seasonal and monthly basis. We explored the importance of year, season and month in explaining smoke pollution via a model selection process. Specifically, we compared combinations of variables using Akaike Information Criterion (AIC) scores; the model with the lowest AIC score considered the model of best fit (Burnham and Anderson 2002). Alternative models with AIC scores within 2 of the best fit model (i.e. ΔAIC ≤ 2) were considered to be equally supported (Burnham and Anderson 2002); any other models not meeting this criterion were considered to have less statistical support We also calculated a KL divergence-based pseudo *R*^2^ for each model as a complementary measure of variance explained (Cameron and Windmeijer 1997). For seasonal analyses, we defined the early dry season as May–July, the late dry season as August–October and the wet season as November–April.

Second, we assessed trends in regional fire activity over the same period. We assessed trends in both (i) annual and seasonal area burnt and (ii) annual and seasonal number of hotspots. Both analyses used a gamma GLM with a log-link function. As with our PM_2.5_ analysis, we used an AIC-based model selection process to determine which combination of variables provided the best model fit. In order to better understand the distribution of fire activity and temporal trends, we conducted two supplementary analyses: (i) an assessment of temporal trends by land tenure category (using a gamma GLM); and (ii) an assessment of geographic patterns in fire activity trends. For the latter, we created a heat map showing the strength and direction of change in fire activity in each landscape cell. We did this for the early and late dry season, and for (a) days associated with exceedances of the national air quality standard (‘exceedance days’) and (b) days not associated with exceedances of the national air quality standard (‘non-exceedance days’). This allowed us to assess whether specific regions were driving changes in early and/or late dry season exceedances. Given that smoke pollution has been shown to be influenced by fire activity in previous days (Price et al. 2012), ‘exceedance days’ were categorised as the day of the exceedance plus the two days leading up to the exceedance, all other days were categorised as non-exceedance days. To create each map, we calculated the average daily number of hotspots for each landscape cell and then used the slope of a linear model predicting the average daily number of hotspots in each cell as a function of year to assign that cell a rate of change in burning between 2004 and 2019.

Third, we investigated the factors influencing smoke pollution in Darwin on a daily basis, in order to inform what might be driving changes over time. Using a gamma GLM with a log-link function, we tested the relative importance of meteorological factors and fire activity on different land tenures in explaining daily PM_2.5_ concentrations. Specifically, we modelled PM_2.5_ concentrations as a function of fire extent (number of hotspots), fire intensity, wind speed and direction, atmospheric stability, and antecedent wet season precipitation. As smoke pollution is affected by fire activity in previous days (Price et al. 2012), our input variables for fire extent and intensity were calculated over the two days leading up to and the day of observation (for full details of all input variables see Table S1). We used an AIC test (Burnham and Anderson 2002) to sequentially subtract variables from a model with all potential variables until we found an optimal (full) model, and calculated a KL divergence-based pseudo *R*^2^ measure of variance explained (Cameron and Windmeijer 1997). We then assessed the relative importance of each variable in predicting smoke pollution in Darwin using the change in model AIC (ΔAIC) and *R*^2^ associated a single-variable model. As with our previous analyses, we used the criterion ΔAIC ≤ 2 as an indicator of equivalence between models. To assess the relative importance of burning on each land tenure category, we replaced the fire extent term (${\mathrm{HS}}_{\mathrm{lag}}$) from across the landscape with ${\mathrm{HS}}_{\mathrm{lag}}$ in each land tenure category separately and reran the model, assessing variance importance as stated above. As a supplementary analysis, we used wind roses to understand differences in wind patterns for the two days leading up to and on the day of exceedances of the PM_2.5_ standard (‘exceedance days’), and on all other days (‘non-exceedance days’) in both the early and late dry seasons.

Finally, we assessed the relationship between net annual and seasonal PM_2.5_ with (i) fire activity (represented by area burnt) and (ii) all other factors supported by analysis outlined above as predictors of daily PM_2.5_ (the Haines Index, sine of wind direction and antecedent precipitation). These analyses used gamma GLMs with a log-link function.

## Results

### Trends in PM_2.5_ pollution

**Fig. 2:**
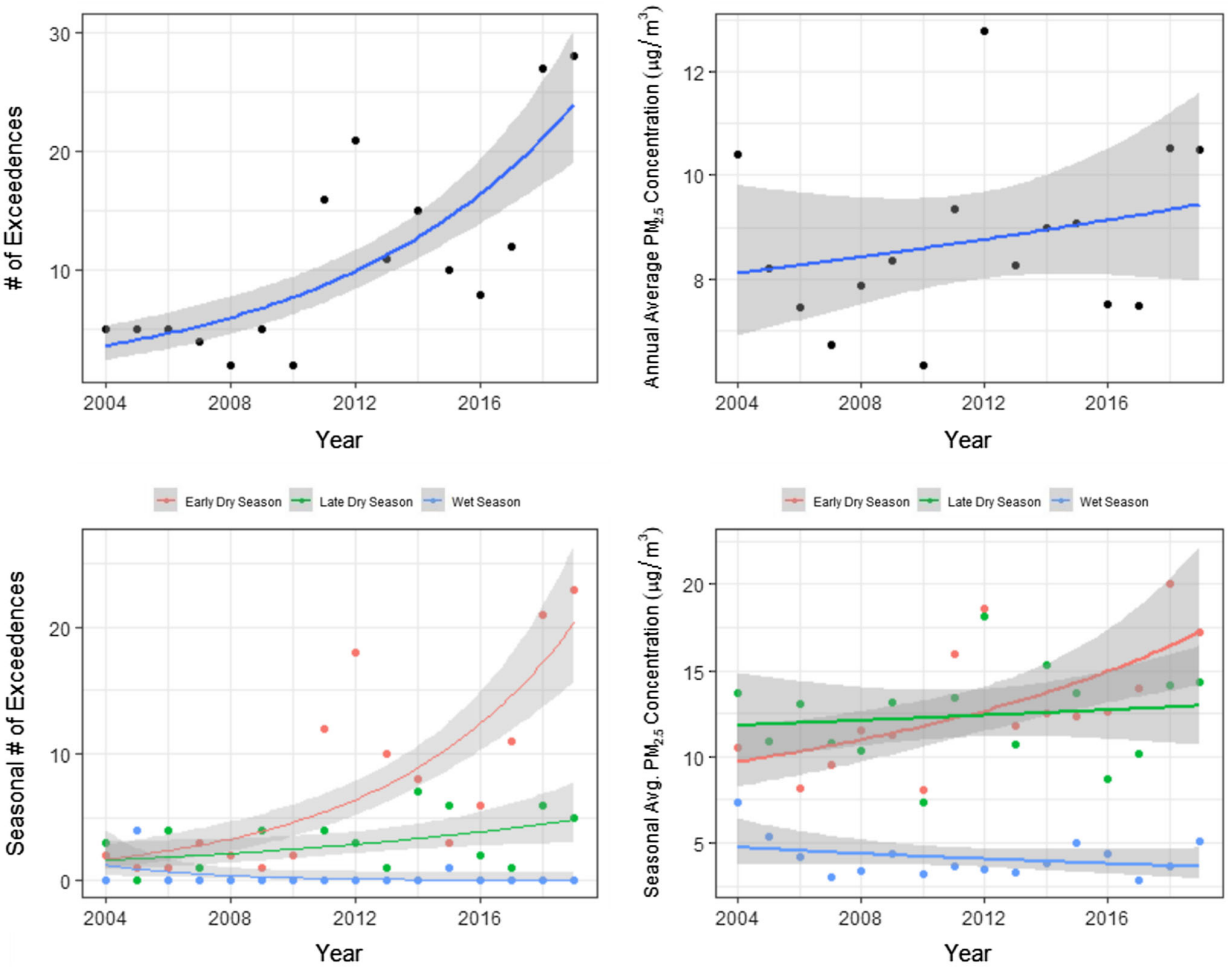
Panel **a** Annual number of days on which there was an exceedance of the national air quality 24-h standard (25 µg/m^3^) at one or more of the active monitoring stations. Panel **b** Annual average PM_2.5_ concentration, where the daily value was based on the maximum daily value across the active monitoring stations. Panel **c** Seasonal number of days on which there was an exceedance of the national air quality 24-h standard (25 µg/m^3^) at one or more of the active monitoring stations. Panel **d** Seasonal average PM_2.5_ concentration, where the daily value was based on the maximum daily average across the active monitoring stations. The trendlines are derived from generalised linear models; the grey ribbon represents one standard error. For **c** and **d**, early dry season = May–July, late dry season = Aug–Oct and the wet season = Nov–April. Statistical details relating to each trend are in Table 1; **a** Model A, **b** Model B, **c** Model C, **d** Model D

Over the period 2004–2019, the data show an increase in the annual number of exceedances of the national 24-h air quality standard for PM_2.5_ (Fig. 2a). The AIC values in Table 1 (Model A) provide strong support that the model with year as a predictor outperforms the null model: the null model has a ΔAIC of 51, supporting the hypothesis that there is an increase in the number of exceedances over time. Further support is provided by a pseudo *R*^2^ of 0.59, indicating that year and exceedances are moderately correlated (Table 1, Model A). The trend in annual average PM_2.5_ concentrations is less clear (Fig. 2b; Table 1, Model B). Here, AIC values suggest the model including year as a predictor performs no better than the null model (ΔAIC ≤ 2, with the null model having the lower AIC), and the proportion of variance explained by year is just 0.07 (Table 1, Model B). Overall, this suggests annual average PM_2.5_ has not increased over time.

**Table 1:** AIC table displaying results for generalised linear models (GLMs) predicting average PM_2.5_ concentration, number of exceedances of the national 24-h PM_2.5_ standard, or area burnt, on the basis of the year and/or season of observation. The model with the most support is listed first, all other models are ranked according to ΔAIC (the difference in Akaike Information Criteria between the best model and given model). *K* is the number of parameters in the model, and the pseudo *R*^2^ represents an estimate of variance explained. The direction of the effect, namely the sign of the coefficient, is also given

	Outcome variables	Predictor variable/s	*K*	AIC	ΔAIC	Log likelihood	Pseudo *R*^2^
Model A	Annual # of exceedances	Year	2	106	0	− 50.5	0.59
		Null model	1	157	51	− 77.3	0
Model B	Annual average PM_2.5_	Null model	2	64.5	0	− 29.8	0
		Year	3	66.5	2	− 29.2	0.07
Model C	Seasonal # of exceedances	Season + Year + Year:Season	6	201	0	− 93.6	0.71
		Season + Year	4	214	13.1	− 102.7	0.65
		Season	3	266	64.4	− 129.5	0.47
		Year	2	350	148.6	− 172.8	0.18
		Null model	1	401	200.1	− 199.6	0
Model D	Seasonal average PM_2.5_	Season + Year + Year:Season	7	211	0	− 96.9	0.84
		Season	4	214	3.8	− 102.8	0.8
		Season + Year	5	216	5	− 102.1	0.81
		Year	3	290	79.6	− 141.8	0.02
		Null model	2	289	78.3	− 142.3	0
Model E	Annual area burnt	Null model	2	517.1	0	− 256.1	0
		Year	3	519	1.9	− 255.5	0.07
Model F	Seasonal area burnt	Season + Year + Year:Season	7	1484	0	− 733.4	0.7
		Season	4	1491	7.2	− 740.9	0.59
		Season + Year	5	1491	7.4	− 739.8	0.61
		Year	3	1532	48.4	− 762.7	0.01
		Null model	2	1530	46.5	− 762.9	0
Model G	Annual average PM_2.5_	Annual area burnt	3	57.3	0	− 24.7	0.47
		Null model	2	64.5	7.1	− 29.8	0
Model H	Seasonal average PM_2.5_	Seas. area burnt + Season + Seasonal area burnt:Season	7	190	0	− 86.8	0.9
		Seas. area burnt + Season	5	196	5.9	− 92.5	0.87
		Season	4	214	24	− 102.8	0.8
		Seas. area burnt	3	254	64	− 124	0.52
		Null model	2	289	98.5	− 142.3	0

When considered by season, strong differences between early and late dry season trends emerge. As shown in Fig. 2c, d, both exceedances and average PM_2.5_ pollution increased strongly in the early dry season (for statistical details see Table 1, Models C and D). In contrast, late dry season exceedances and PM_2.5_ concentrations recorded only a very slight increase (Fig. 2c, d). In the wet season, both air pollution metrics showed little change. For both exceedances and PM_2.5_ concentrations, the model including an interaction between season and year out-performed both the null and all simpler models, suggesting the combination of seasonal and annual trends best explains Darwin’s air pollution over our study period (Table 1, Models C and D).

On a monthly basis, the most substantive increases in the number of exceedances occurred in June and July (see Fig. S3). There were less pronounced increases in May and August and no increases in September and October.

### Trends in fire activity

**Fig. 3:**
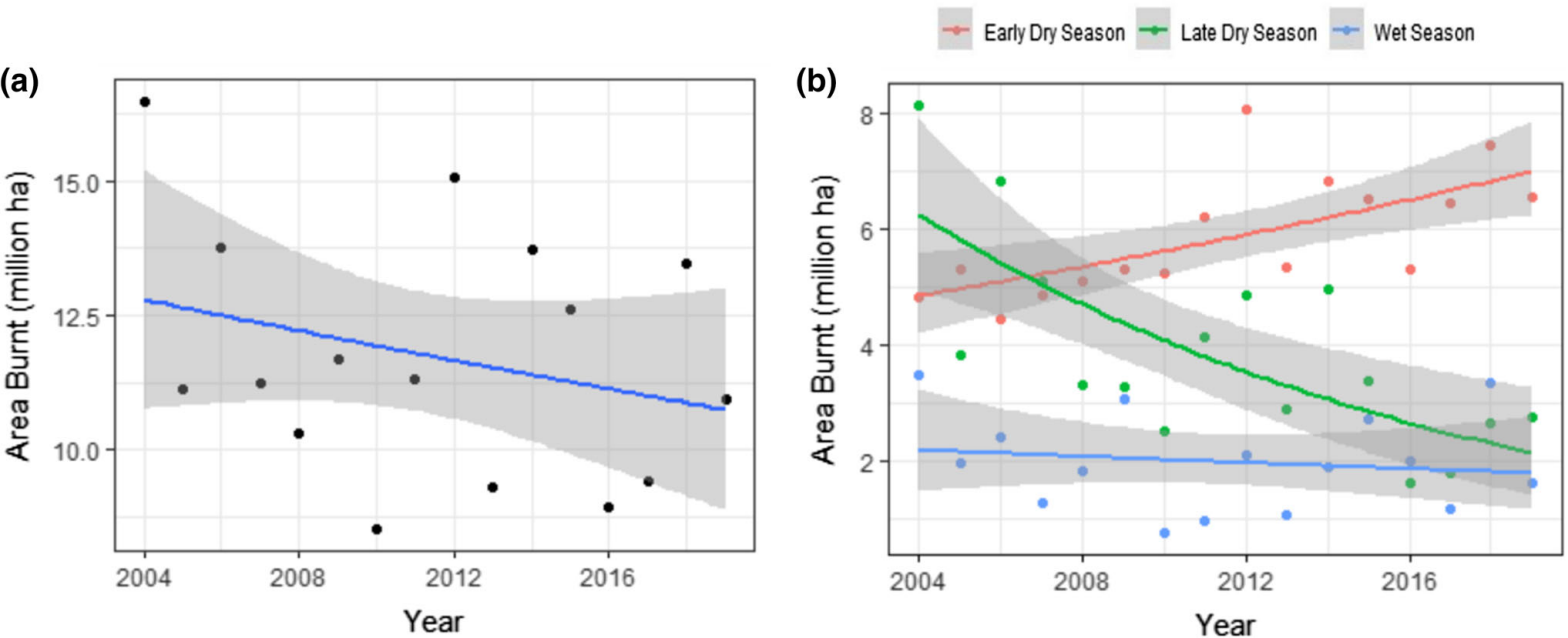
Temporal trends in area burnt within a 500 km radius of Darwin on an annual (**a**) and seasonal (**b**) basis. Data cover the period 2004–2019. Trendlines are derived from generalised linear models; the grey ribbon represents one standard error. For **b** early dry season = May–July, late dry season = Aug–Oct and the wet season = Nov–April. For statistical details see Table 1

There is no clear trend in net annual area burnt within a 500 km radius of Darwin. As shown in Fig. 3a, there appears to be a slight decrease in net annual area burnt over the 2004–2019 study period, however there is a significant amount of scatter and the trend is not supported by AIC or pseudo *R*^2^ values (Table 1, Model E). Once again, clearer trends emerge when broken down by season. As shown in Fig. 3b, there was a clear increase in area burnt in the early dry season and a decrease in area burnt in the late dry season. Here, AIC values provide strong support for the interaction of year and season; this combination of variables outperformed the null and all simpler models. This indicates statistical support for different trends in different seasons (for full details, see Table 1, Model F). Trends in hotspots were very similar, see Fig. S4.

**Fig. 4:**
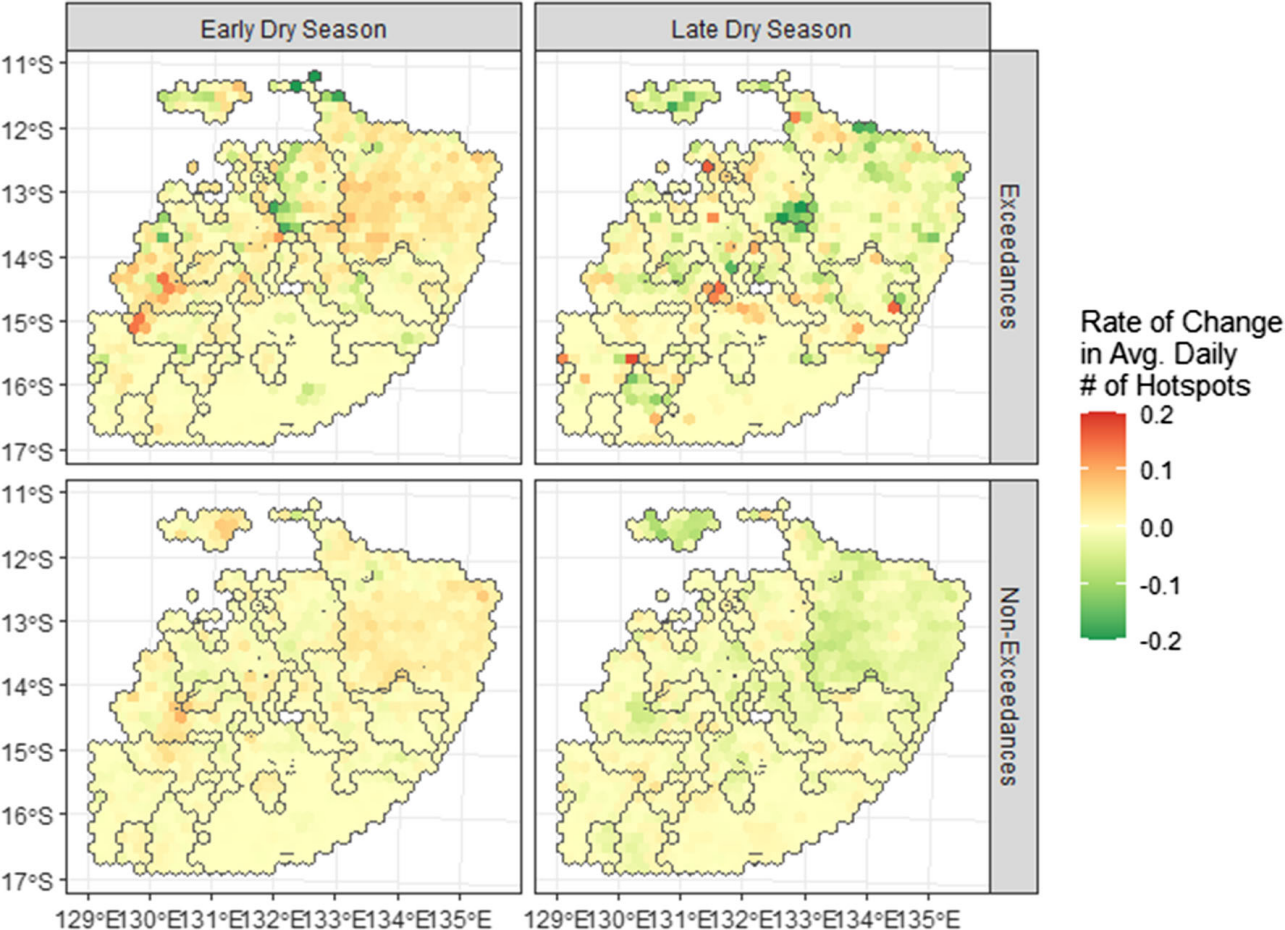
Heat map of change in fire activity in the study area by season. The map specifically shows the geographic distribution of changes in fire activity between 2004 and 2019, on days where the air quality exceeded the national air quality standard (‘exceedances’), and on days when it did not (‘non-exceedances’). Rate of change was calculated using the slope of a linear model predicting average daily number of hotspots per grid cell as a function of year. Colouring indicates the rate of change as indicated, with red representing increases in fire activity, and green representing decreases. Black outlines represent locations of tenure class categories as indicated in Fig. S1 for reference

Our geographic analysis found clear spatial and land tenure-based patterns in fire activity trends. As shown in Fig. 4, the strongest increases in the number of hotpots in the early dry season were observed in two areas: first, the region east of Darwin, particularly the Arnhem Land Plateau, and second, the region to Darwin’s south and south-west. These areas also recorded the strongest decreases in the number of hotspots recorded in the late dry season. Trends were stronger on exceedance compared to non-exceedance days. When considered by land tenure, the most substantial increases in early dry season fire activity have been in Arnhem Land and on other Aboriginal Land (see Fig. S5). On protected lands (whether Commonwealth or Northern Territory Government) and pastoral land, there was negligible change.

### Daily PM_2.5_ pollution drivers

Our statistical modelling indicated that daily fire activity has a much stronger association with smoke pollution in Darwin than any meteorological factor. The optimal model for daily PM_2.5_ pollution included fire activity, antecedent precipitation, sine of wind direction, and the Haines Index; however, when each variable was considered independently, the landscape-wide number of hotspots recorded in the previous 2-day period was clearly the strongest predictor of daily smoke pollution, explaining 39% of the variation (pseudo *R*^2^ = 0.39, full details in Table 2). By comparison, the effect of meteorology on smoke pollution in Darwin was insubstantial. Although the antecedent wet season’s precipitation, wind direction and atmospheric stability all had some support as predictors (based on AIC values at least 2 lower than the null model), they explained extremely small amounts of variance (pseudo *R*^2^ ≤ 0.05 in all cases, see Table 2).

**Table 2:** AIC table displaying results for a gamma generalised linear model (GLM) predicting daily average PM_2.5_ concentration from fire activity and weather. To assess relative importance, single-variable models were compared with the model with the most support (first model listed). Models are ranked according to ΔAIC (the difference in Akaike Information Criteria between the best model and given model). *K* is the number of parameters in the model, and the pseudo *R*^2^ represents an estimate of variance explained. $\sqrt[3]{{\mathbf{H}\mathbf{S}}_{\mathbf{l}\mathbf{a}\mathbf{g}}}$ represents the number of hotspots on lag days (the 2 days leading up to and the day of the PM_2.5_ concentration measurement), and ${\mathbf{FRP}}_{\mathbf{lag}}/ {\mathbf{HS}}_{\mathbf{lag}}$ is a measure of the intensity of those hotspots. The direction of the effect, namely the sign of the coefficient, is also given for each individual predictor variable

Variables	Direction of effect	*K*	AIC	ΔAIC	Log likelihood	Pseudo *R*^2^
Full Model		7	20 728	0	− 10 357.1	0.42
$\sqrt[3]{{\mathbf{H}\mathbf{S}}_{\mathbf{l}\mathbf{a}\mathbf{g}}}$: Total	+	3	20 924	195.4	− 10 458.8	0.39
${{\mathbf{F}\mathbf{R}\mathbf{P}}_{\mathbf{l}\mathbf{a}\mathbf{g}}}/{{\mathbf{H}\mathbf{S}}_{\mathbf{l}\mathbf{a}\mathbf{g}}}$	+	3	22 604	1875.8	− 11 298.9	0.05
Antecedent Precipitation	+	3	22 636	1907.9	− 11 315	0.04
Sine of Wind Direction	−	3	22 693	1964.5	− 11 343.3	0.03
Haines Index	+	3	22 739	2010.5	− 11 366.3	0.01
Null Model		2	22 792	2063.4	− 11 393.8	0

Notably, however, our wind rose analysis found that exceedances of the national air quality standard were strongly associated with winds from the south-east quadrant (Fig. S6). This is the prevailing wind direction in the early dry season, when the trade winds blow (Fig. S7). Almost all early dry season exceedances were associated with winds from this south-east quadrant (Fig. S7). In the late dry season, wind direction is more variable but the prevailing winds are onshore from the north–north-west (Fig. S7). In the late dry season, exceedance days included a disproportionate representation of days with winds from the east; however winds from a wider range of directions were associated with exceedances of the air quality standard (Fig. S7).

When considered by land tenure, daily PM_2.5_ pollution in Darwin has the strongest relationship with hotspots on Aboriginal land other than Arnhem Land (*R*^2^ = 0.35), followed by Kakadu (*R*^2^ = 0.21), Arnhem Land (*R*^2^ = 0.2) and pastoral land (*R*^2^ = 0.16, full details in Table S2).

### Drivers of annual and seasonal PM_2.5_ pollution trends

**Fig. 5:**
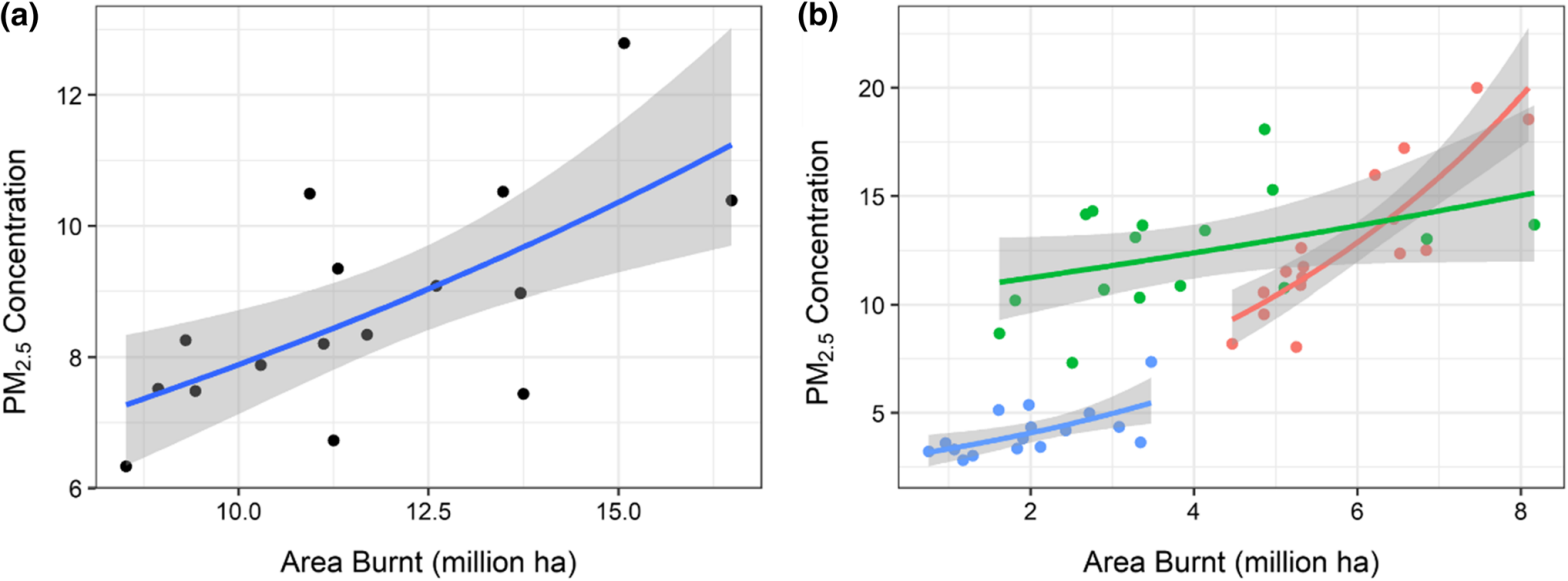
The relationship between average PM_2.5_ concentrations in Darwin and area burnt within a 500 km radius on an annual (**a**) and seasonal (**b**) basis. Early dry season is defined as May–July, the late dry season as Aug–Oct and the wet season (Nov–April). Data cover the period 2004–2019. Lines represent trends derived from generalised linear models, the grey shading represents one standard error. For statistical details see Table 1

Our analysis of the relationship with PM_2.5_ pollution and fire activity on an annual and seasonal basis provided strong evidence that the relationship between daily smoke pollution and fire activity extends to annual and seasonal time scales. As shown in Fig. 5a and Table 1 (Models G and H), the relationship between area burnt and both annual and seasonal average PM_2.5_ concentrations was positive and supported by AIC and pseudo *R*^2^ values. On a seasonal basis, the slope of the relationship is steepest in the early dry season; indicating that early dry season burning has a stronger association with PM_2.5_ concentrations in Darwin than late dry or wet season fire activity. There is statistical support for a substantial difference between seasons in the relationship between area burnt and PM_2.5_ concentrations (Table 2, Model H).

Our analysis of the relationship between annual and seasonal PM_2.5_ pollution and meteorological factors found no support for a relationship between meteorological factors and early dry season PM_2.5_ (Table S3). In contrast, a relationship between meteorology and late dry season PM_2.5_ concentrations was supported; here, the full model including all three meteorological factors as predictor variables had the strongest statistical support (Table S3). Wind direction explained the greatest amount of variation as an independent predictor, with a pseudo *R*^2^ of 0.52 (Table S3). In the wet season, Haines Index appeared to be the strongest predictor of PM_2.5_. On an annual basis, there is no clear relationship between average PM_2.5_ and any meteorological variable (Table S3).

## Discussion

We have shown that between 2004 and 2019, air quality has worsened in Darwin in the early dry season (particularly the months of June–July), and has not changed substantially in other seasons. Trends in smoke pollution are very closely linked to seasonal trends in fire activity in at least a 500 km radius of Darwin; and notably, the relationship between fire activity and smoke pollution in Darwin is strongest in the early dry season, when the south-easterly trade winds blow. Exceedances of the national air quality standards are strongly associated with winds from this quadrant; and in the late dry season, seasonal average PM_2.5_ was higher in seasons with a greater proportion of winds from the east–south-east. The implications of these results highlight some complex trade-offs and considerations with respect to landscape-scale savanna burning programs, both within and beyond our northern Australian study context.

First, given the correlation between greenhouse gas and particulate emissions (van der Werf et al. 2017), our findings are surprising, given the expectation that early season burning reduces greenhouse gas emissions. Savanna burning for carbon abatement is premised on the assumption that less intense and/or extensive early dry season burning reduces more intense and/or extensive late dry season burning, and thus has a net positive impact on annual greenhouse gas, and presumably particulate, emissions (Russell-Smith et al. 2015).

Our findings highlight that the story is more complex with respect to particulate emissions and/or population exposure: despite a substantial expansion of savanna burning for carbon abatement over our study period, net annual PM_2.5_ concentrations did not decline, and the number of exceedances of the national air quality standard increased. Part of this apparent conflict can be explained by the nexus of meteorology and the location prescribed early dry season burning in relation to population centres. Our findings indicate that one important driver of worsening early dry season particulate pollution is the coincidence of large areas of savanna being burned for carbon abatement to the east–south-east of Darwin in the early dry season, when there are steady south-easterly trade winds (see Figs. S7, S8). Wind directionality may also explain the stronger relationship between fire activity on Aboriginal as compared to other tenures of land, given large areas are upwind of the prevailing south-easterly trade winds.

Fuel dynamics may be another important consideration. It is also possible that the ongoing expansion of highly flammable native and non-native grasses has rendered landscapes more prone to extensive and/or intense fires in the early dry season, reducing the smoke emissions benefits of early dry season burning. There is evidence that annual sorghum (*Sarga* sp.), which cures early in the dry season, has become dominant on frequently burned *Eucalyptus* savannas, driving a grass-fire cycle (Bowman et al. 2014). Additionally, the invasive exotic grass species, *Andropogon gayanus* (gamba grass) has become established in the savannas surrounding Darwin, increasing fuel loads by up to seven times compared to native grass-dominated savanna, resulting in fires with up to eightfold higher intensity (Rossiter et al. 2003). Overall, these fuel-related trends have the potential to substantially impact fire activity and air pollution trends and should be carefully considered in the context of carbon abatement programs. It is also possible that climate change is affecting the north Australian savannas in ways that reduce fuel moisture in the early dry season: for example by altering rainfall totals, seasonal rainfall patterns, and by increasing temperatures, noting that we were unable to investigate this hypothesis because the very high interannual variability of rainfall in the Australian monsoon tropics (Harris and Lucas 2019) makes it difficult to detect trends in fire weather from short record length (Williamson et al. 2016b).

Overall, our analysis of smoke pollution and fire activity over this fire-prone region, based on the integration of remote sensing, meteorological, geographic and air quality data over 15 years, has enabled us to highlight patterns, trends and relationships between fire activity and smoke pollution originating from different directions and land tenures. We acknowledge we have not undertaken detailed back-trajectory analyses and are thus only able to draw broad inferences about the geographic sources of smoke over Darwin. Notwithstanding these limitations, these data clearly demonstrate that Darwin’s already significant air quality problem is worsening, rather than improving, in association with increased early dry season burning. The clear implication for policy makers locally and globally is that population exposure to smoke pollution cannot be assumed as a co-benefit of savanna burning for carbon abatement: to the contrary, care must be taken to ensure there are not unintended negative smoke exposure consequences.

In this context, policy levers may need to consider how to regulate burning to avoid increased health impacts associated with smoke pollution exposure. In Darwin’s specific context, particular attention may be needed in locations to the east–southeast of the city from whence the trade winds blow.

One solution, in Darwin or elsewhere, may be a coordinated smoke management system that works across multiple land managers to regulate the amount of smoke that can be released into a specific airshed on a given day. In flammable landscapes it is increasingly recognised that prescribed burning plans must be integrated with smoke management (Hardy et al. 2001). For example, in Tasmania, land managers participate in a bidding system for the right to conduct prescribed burning, based on burn location, predicted smoke emissions, and meteorological conditions. In this way the system aims to cap the amount of smoke released into a specific airshed on a given day and is considered to be generally effective (Chuter 2011). Such a scheme could be applied to cap the amount of smoke released per day from locations where pollution will be directed towards Darwin and/or other target populations. We assert it is a reasonable policy response to regulate burning to offset the impacts of smoke pollution which carry quantifiable health costs (Borchers Arriagada et al. 2020): especially given that prescribed burning is increasingly undertaken to earn carbon credits for land owners, leading to potential inequity in the distribution of benefits and costs.

In this context, given the significant human health threat posed by smoke from savanna fires (Johnston et al. 2012), our research highlights the importance of understanding the trade-offs between prescribed burning and human exposure to particulate pollution across flammable environments, such as tropical savannas, around the globe. Of particular importance is understanding the relative health costs compared to the income generated from carbon offsets associated with prescribed tropical savanna burning programs. Given the likely continued expansion of savanna burning for carbon abatement, both in Australia and beyond, further research to inform program designs that minimise health-carbon abatement trade-offs, would be highly valuable.

## Conclusion

Our geospatial analysis has identified that early dry season burning is associated with worsening air quality in Darwin, the capital of Australia’s Northern Territory. The cause of the increased pollution appears related to the combination of large areas of tropical savanna to the southeast of Darwin being intentionally burned in the early dry season when south-easterly trade winds prevail. A driver for the increased early dry season burning in this region has been the introduction of a carbon abatement scheme. Given the demonstrable human health impacts of biomass smoke air pollution, our study highlights the need to more fully understand the trade-offs of prescribed burning schemes designed to generate income through carbon abatement, and develop solutions that minimise unintended impacts on human health.

## Supplementary Information

Below is the link to the electronic supplementary material.

Supplementary file1 (PDF 3297 kb)
