# Supplementary material for: Smoke pollution must be part of the savanna fire management equation: A case study from Darwin, Australia
Source: Ambio. 2022 May 24;51(11):2214–26. doi: 10.1007/s13280-022-01745-9 (PMC9481847; doi:10.1007/s13280-022-01745-9)
Supplement: Supplementary file 1 — Supplementary file1 (PDF 3297 kb) [file 13280_2022_1745_MOESM1_ESM.pdf]

Ambio

Supplementary Information

This supplementary information has not been peer reviewed.

Title: **Smoke pollution must be part of the savanna fire management equation: a case study from Darwin, Australia**

Table S1. Definitions of input variables for a Gamma Generalised Linear Model testing the relative importance of fire activity and other factors in explaining daily $PM_{2.5}$ concentrations in Darwin.

**Variable**	**Calculation**
Cumulative fire extent over the two days leading up to and the day of observation	Number of daytime MODIS hotspots within 500 km of Darwin over the two days preceding and the day of the observation (HS_{lag}). The model used the cubed root, $\sqrt[3]{HS_{lag}}$ as this was more correlated with $PM_{2.5}$ than the untransformed variable.
Cumulative fire intensity over the two days leading up to and the day of observation	Sum of Fire Radiative Power for all MODIS hotspots incorporated in the above. The model used FRP_{lag}/HS_{lag} in order to avoid multicollinearity.
Wind speed	Wind speed in km hr^{-1}
Wind direction	Sine and cosine transformations of wind direction
Atmospheric stability	Continuous Haines index
Antecedent rainfall	Antecedent wet season (Nov-April) precipitation over all areas with hotspots recorded in the two days leading up to and of the observation

Table S2. Expanded version of Table 2, displaying results for a Gamma Generalised Linear Model (GLM) predicting daily average $PM_{2.5}$ concentration from fire activity and weather. Single variable models for daily lagged fire activity on each of seven land tenure classes are compared to the models listed in Table 2. Note that these variables (in italics) were not included in the full model. Models are ranked using ΔAIC, with the best performing model (in this case the full model) being the baseline for ΔAIC calculation. K is the number of parameters in the model, and the pseudo R^2 represents an estimate of variance explained.

Predictor Variable	K	AIC	ΔAIC	Log Likelihood	Psuedo R^2
Full model	7	20728	0	-10357.1	0.42
$\sqrt[3]{\boldsymbol{HS_{lag}}}$: Total	3	20924	195.4	-10458.8	0.39
$\sqrt[3]{\boldsymbol{HS_{lag}}}$: *Other Aboriginal Land*	3	21192	463.4	-10592.8	0.35
$\sqrt[3]{\boldsymbol{HS_{lag}}}$: *Commonwealth Parks (Kakadu)*	3	21888	1159.4	-10940.8	0.21
$\sqrt[3]{\boldsymbol{HS_{lag}}}$: *Arnhem Land*	3	21952	1223.8	-10973	0.2
$\sqrt[3]{\boldsymbol{HS_{lag}}}$: *Pastoral Land*	3	22138	1410.1	-11066.1	0.16
$\sqrt[3]{\boldsymbol{HS_{lag}}}$: *Other Land*	3	22153	1425.2	-11073.7	0.15
$\sqrt[3]{\boldsymbol{HS_{lag}}}$: *NT Conservation Areas*	3	22333	1604.9	-11163.5	0.11
$\boldsymbol{FRP_{lag}}/\boldsymbol{HS_{lag}}$	3	22604	1875.8	-11298.9	0.05
Antecedent Precipitation	3	22636	1907.9	-11315	0.04
Sine of Wind Direction	3	22693	1964.5	-11343.3	0.03
$\sqrt[3]{\boldsymbol{HS_{lag}}}$: *Military Land*	3	22710	1982.2	-11352.2	0.02
Haines Index	3	22739	2010.5	-11366.3	0.01
Null Model	2	22792	2063.4	-11393.8	0

Table S3 AIC table displaying results for generalised linear models (GLM) predicting annual average $PM_{2.5}$ concentration for each season as a function of three different meteorological variables (as visualised in Fig. S8). For each season, a full model was compared to single variable models to assess explanatory power. Models are ranked using ΔAIC, with the optimal model (lowest AIC) listed first and used as the ΔAIC baseline.

Outcome variable	Predictor Variables	K	AIC	ΔAIC	Log Likelihood	Pseudo-R^2
Early Dry Season average $PM_{2.5}$	Intercept Only (Null)	2	82	0	-38.3	0
Early Dry Season average $PM_{2.5}$	Antecedent Precipitation	3	83	1.9	-37.6	0.08
Early Dry Season average $PM_{2.5}$	Haines Index	3	84	2.5	-37.9	0.04
Early Dry Season average $PM_{2.5}$	Sin(Wind Direction)	3	85	3	-38.2	0.01
Early Dry Season average $PM_{2.5}$	Full: Haines + Wind + Precipitation	5	90	8	-36.4	0.22
Late Dry Season average $PM_{2.5}$	Full: Haines + Wind + Precipitation	5	66	0	-24.7	0.77
Late Dry Season average $PM_{2.5}$	Sin(Wind Direction)	3	69	2.8	-30.3	0.52
Late Dry Season average $PM_{2.5}$	Haines Index	3	71	4.9	-31.4	0.45
Late Dry Season average $PM_{2.5}$	Intercept Only (Null)	2	77	10.7	-35.8	0
Late Dry Season average $PM_{2.5}$	Antecedent Precipitation	3	77	11	-34.4	0.17
Wet Season average $PM_{2.5}$	Haines Index	3	37	0	-14.3	0.6
Wet Season average $PM_{2.5}$	Full: Haines + Wind + Precipitation	5	40	3.1	-11.6	0.72
Wet Season average $PM_{2.5}$	Sin(Wind Direction)	3	47	10.6	-19.5	0.2
Wet Season average $PM_{2.5}$	Intercept Only (Null)	2	47	10.7	-21.2	0
Wet Season average $PM_{2.5}$	Antecedent Precipitation	3	50	13.4	-21	0.03
Annual average $PM_{2.5}$	Haines Index	3	63	0	-27.4	0.25
Annual average $PM_{2.5}$	Sin(Wind Direction)	3	64	1.2	-28.1	0.19
Annual average $PM_{2.5}$	Intercept Only (Null)	2	64	1.6	-29.8	0
Annual average $PM_{2.5}$	Full: Haines + Wind + Precipitation	5	65	2.6	-24.7	0.47
Annual average $PM_{2.5}$	Antecedent Precipitation	3	68	4.7	-29.8	0

(a)

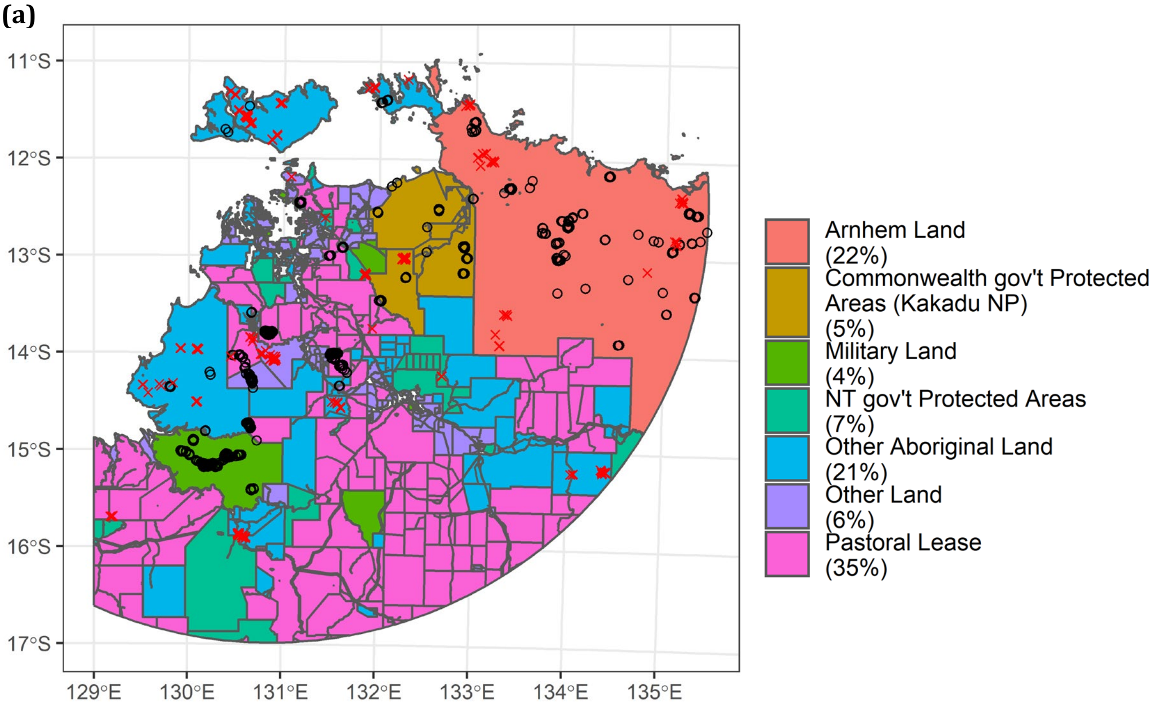

(b)

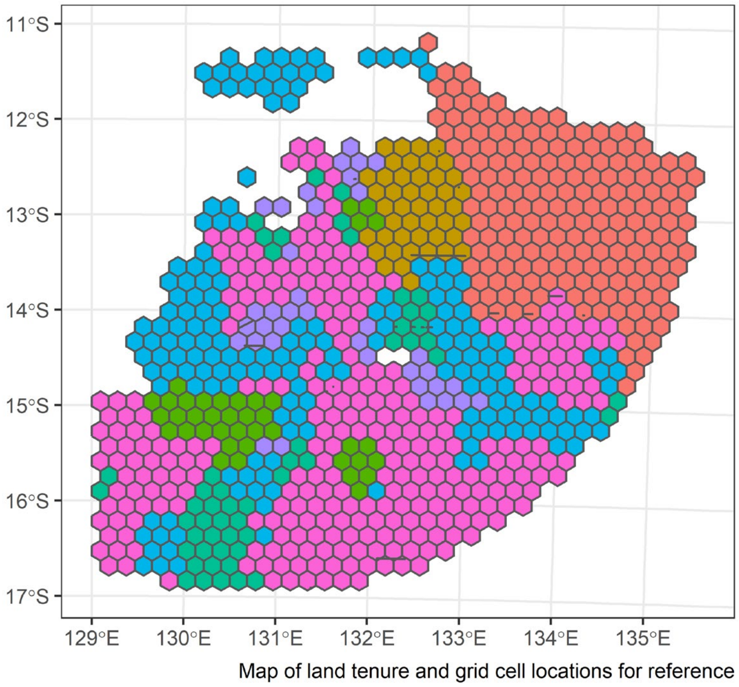

Fig. S1: Map of geospatial data used for this study. The parcels in panel (a) represent the location of broad land-tenure categories within the study area, with the locations of a set of hotspots for visualisation purposes. The black circles represent hotspots detected on 6 July 2019 (when daily average $PM_{2.5}$ concentration was 62.5 µg m^{-3}), whereas red crosses represent hotspots detected on 25 July 2005 (when daily average $PM_{2.5}$ concentration was 11.5 µg m^{-3}). Panel (b) represents our grid simplification approach, where we divided the study area into 845 x 20 km hexagonal grid cells. We assigned a land tenure class to each cell based on the class that occupied the most area within each cell.

(a)

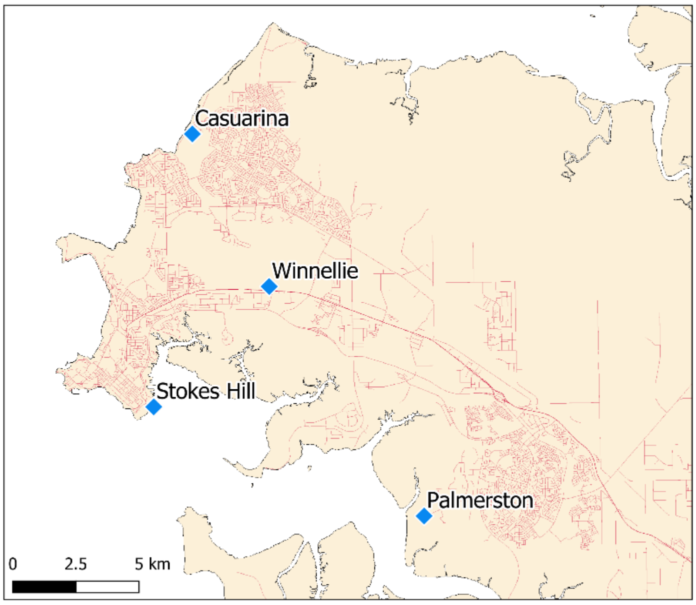

(b)

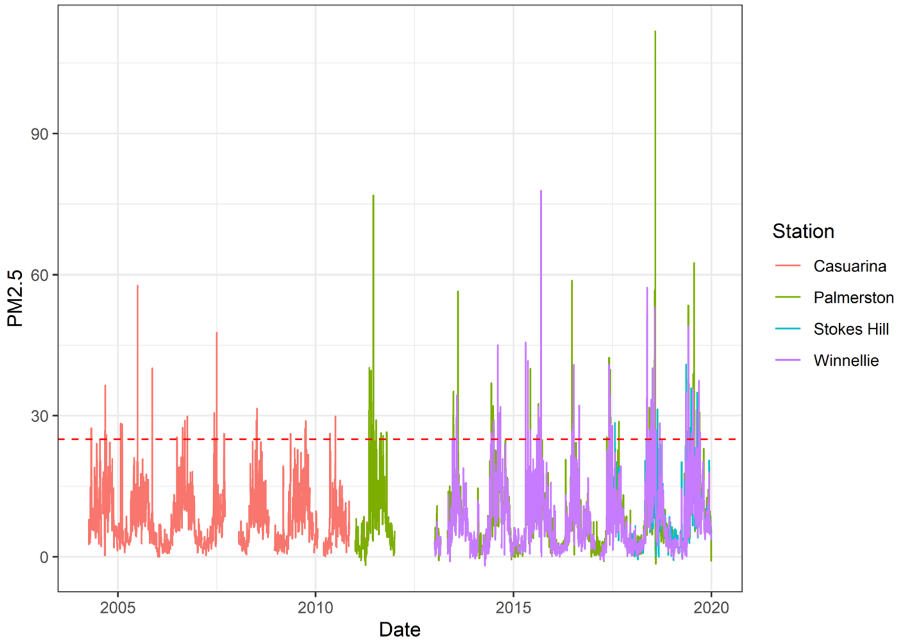

Fig. S2: Panel (a) Location of the four monitoring stations which provided $PM_{2.5}$ data for this study. The stations are operated by the Northern Territory Environment Protection Authority. Casuarina operated from April 2004-December 2011, Palmerston from January 2011-December 2019, Stokes Hill from July 2017-December 2019, and Winnellie from January 2013-December 2019. Panel (b) Daily average $PM_{2.5}$ concentration ($\mu g/m^3$) from the four stations from 2004-2019. Colour coding indicates the station; the red dashed line indicates the national 24-hour standard (25 $\mu g/m^3$).

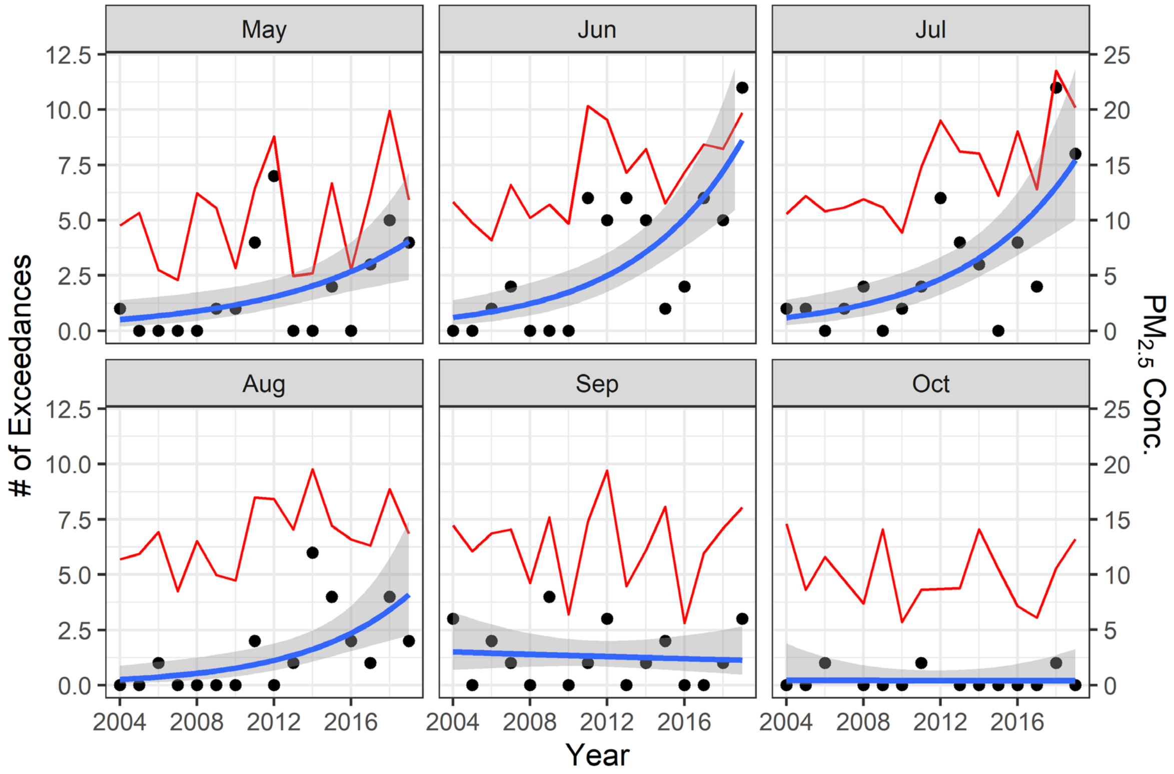

Fig. S3. Monthly number of exceedances (black points; left y-axis), and monthly average $PM_{2.5}$ concentration (red lines; right y-axis), in Darwin from 2004-2019. Only months during the dry season are shown as there is little $PM_{2.5}$ pollution during the wet season months. Blue trendlines represent the trends from a Generalised Linear Model.

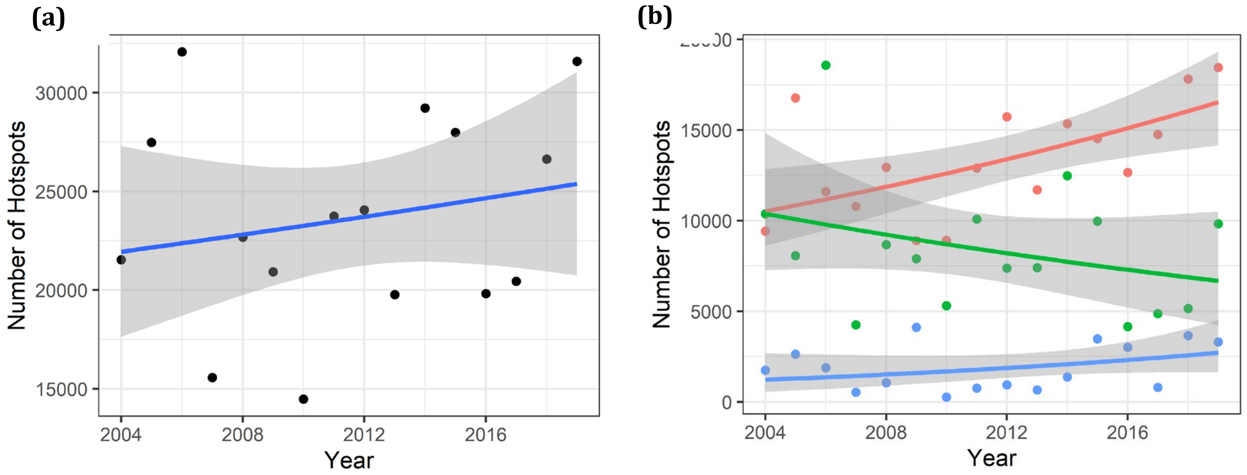

Fig. S4. Temporal trends in the number of hospots observed within a 500 km radius of Darwin on an annual (a) or seasonal (b) basis. Data cover the period 2004-2019; hotspots were determined on the basis of MODIS thermal anomaly data (NASA FIRMS, 2020). Trendlines are derived from Generalised Linear Models; the grey ribbon represents one standard error. For (b), early dry season = May-Jul, late dry season = Aug-Oct and the wet season = Nov-Apr.

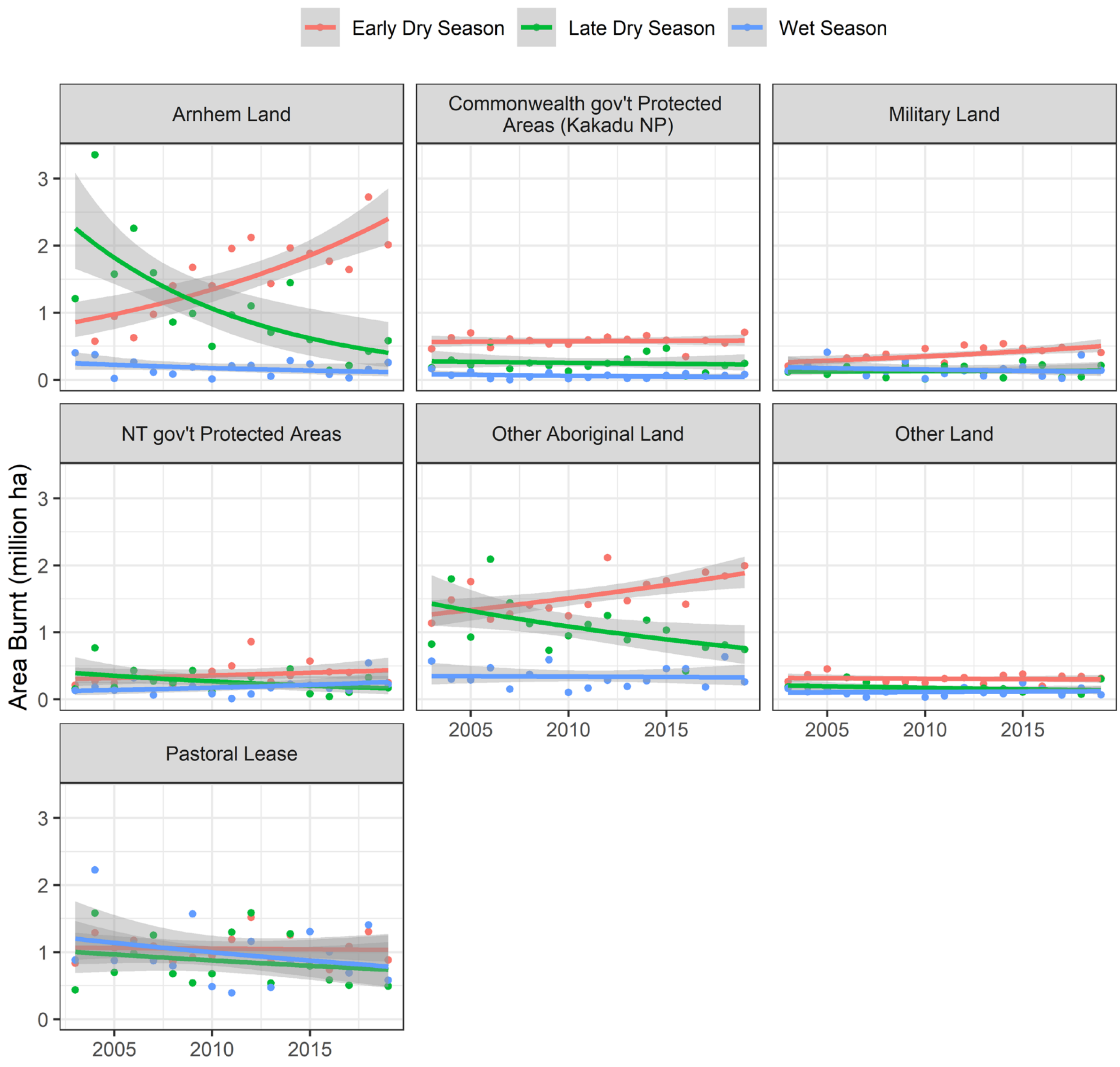

Fig. S5: Temporal trends in area burnt by land tenure category and season. Lines represent trends derived from Generalised Linear Models, and ribbons represent one standard error. Lines and points are coloured based on season as indicated, early dry season = May-Jul, late dry season = Aug-Oct and the wet season = Nov-Apr.

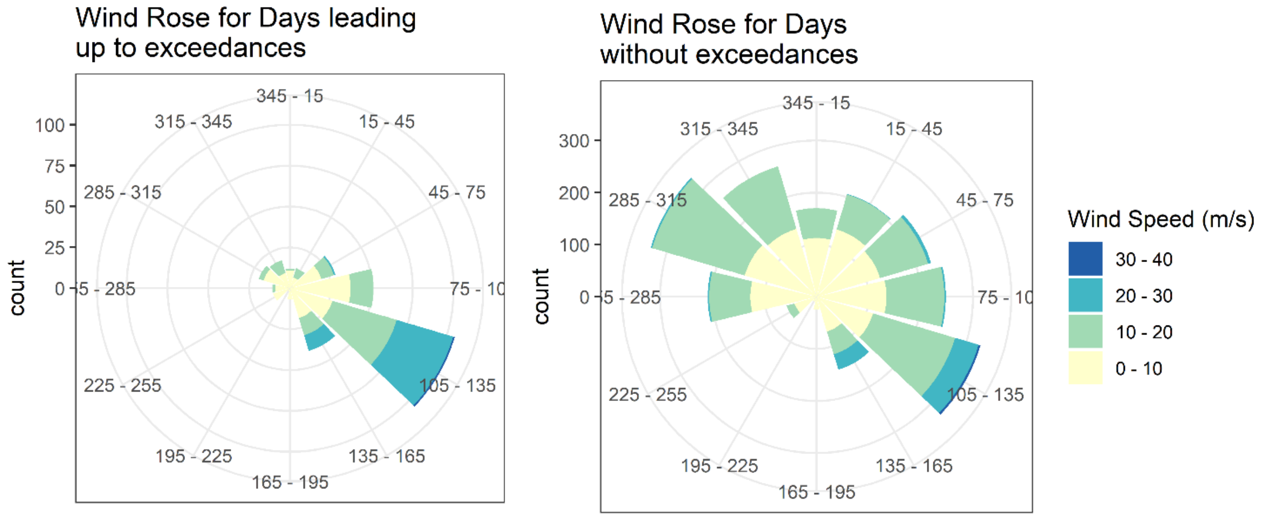

Fig. S6: Wind speed and direction on exceedance days vs non-exceedance days. Wind Rose describing the daily average direction and speed of wind on (a) the two days leading up to and on the day of an air quality exceedance ('exceedance days') and (b) on all other days ('non-exceedance days'). The radial axis represents the number of observations in each category. Note the count axes are not the same due to the larger overall number of days in panel (b); the plot pair simply demonstrates the difference in proportion of days with winds of a particular speed and direction on exceedance vs non-exceedance days.

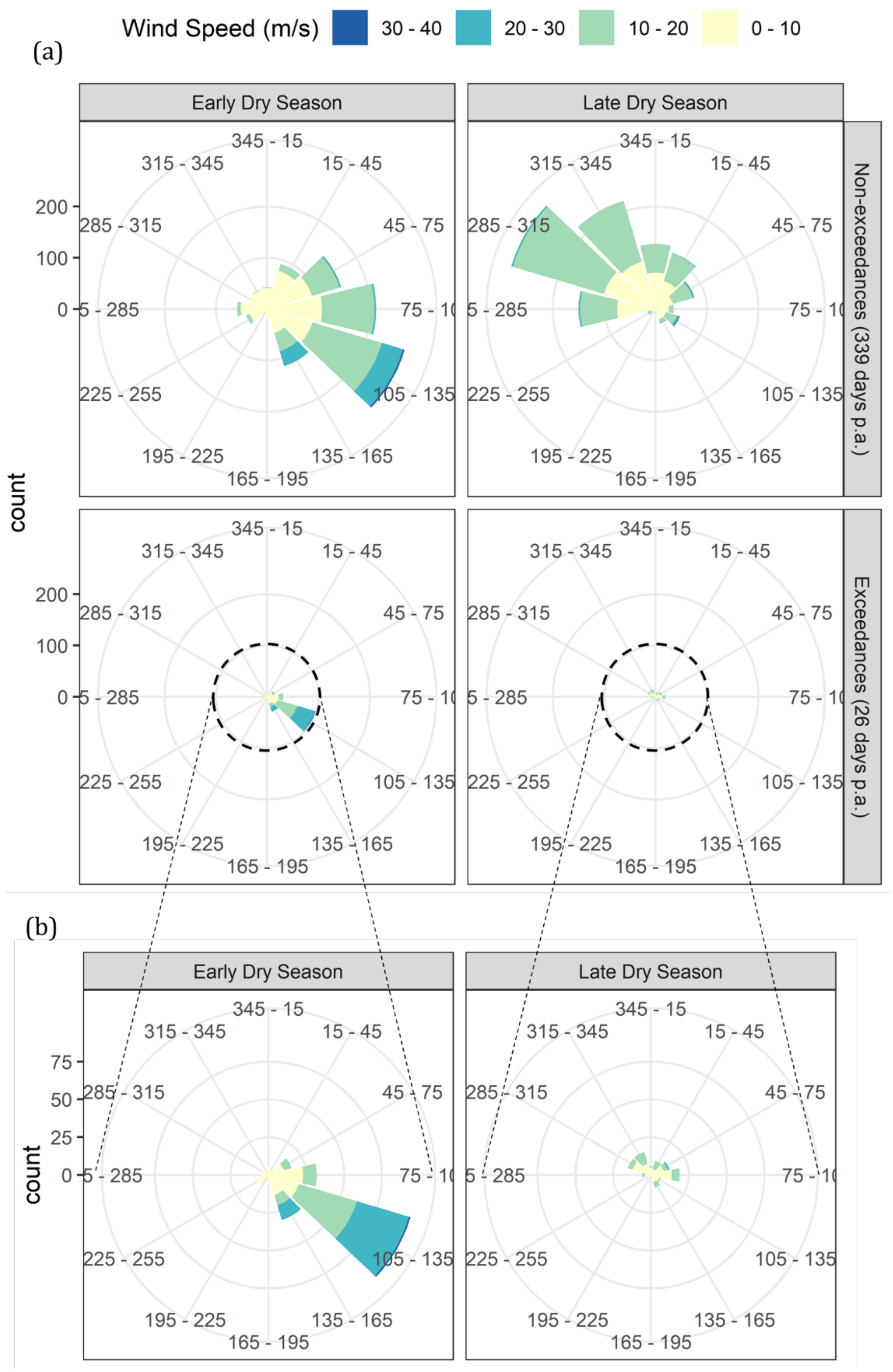

Fig. S7: Wind speed and direction on exceedance and non-exceedance days by Season. Wind Roses describing the daily average direction and speed of wind, separated by season (top panel), on two days leading up to and on the day of air quality exceedances (Exceedances) and on all other days (Non-exceedances). The radial axis represents the number of observations in each category. (a) compares observations on exceedance and non-exceedance days using a common radial axis scale, whereas (b) displays observations on exceedance days using an altered scale for readability.

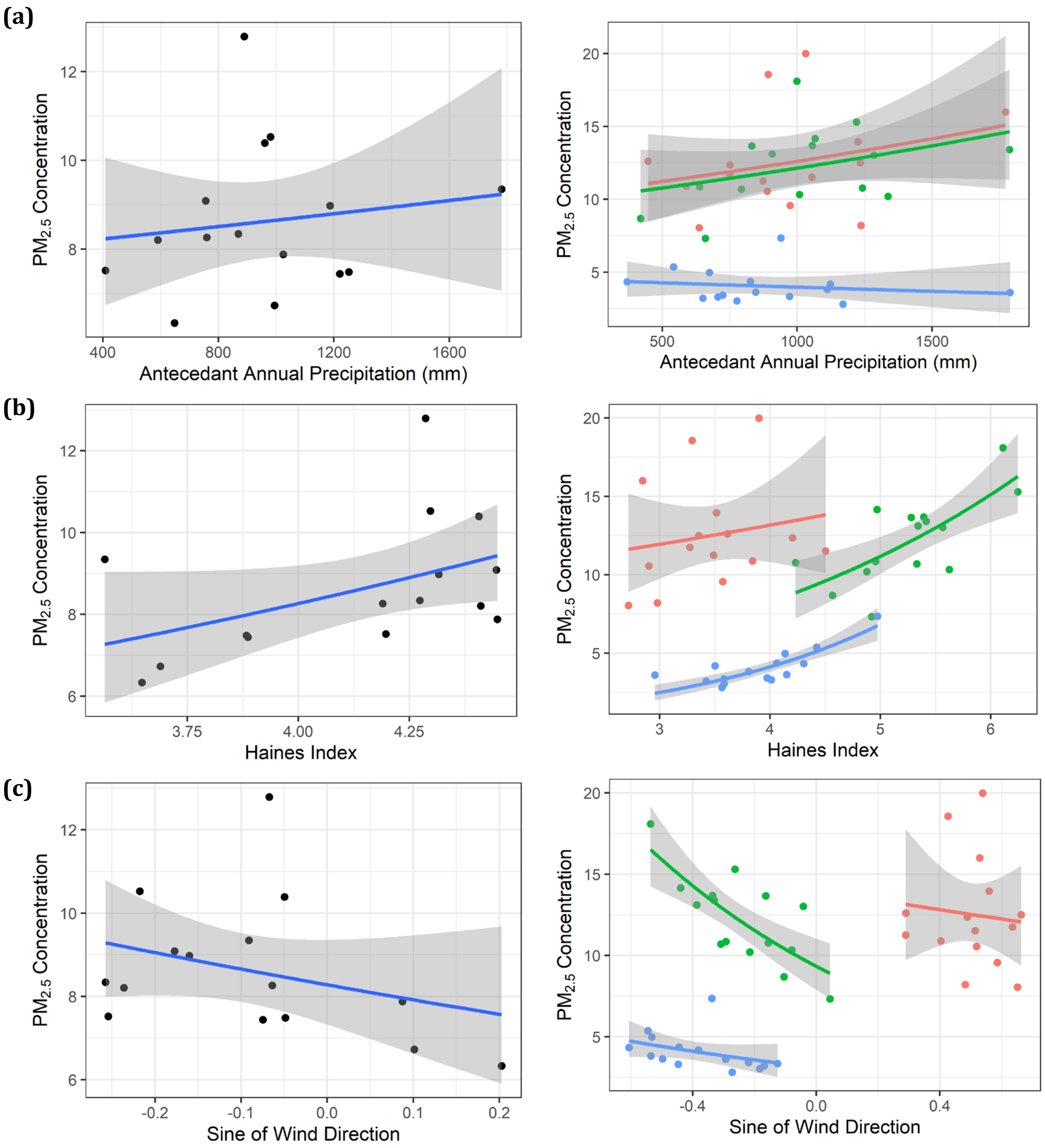

Fig. S8. The relationship between annual and seasonal average $PM_{2.5}$ concentrations in Darwin and (a) antecedent wet season precipitation, (b) the Haines Index, and (c) sine of wind direction (east-west directionality). Early dry season is defined as May-Jul, the late dry season as Aug-Oct and the wet season as Nov-April. Data cover the period 2004-2019. Lines represent trends derived from Generalised Linear Models, the grey shading represents one standard error.
